# A single-armed proof-of-concept study of Lymfit: A personalized, virtual exercise intervention to improve health outcomes in lymphoma survivors in the pandemic

**DOI:** 10.1371/journal.pone.0275038

**Published:** 2024-01-05

**Authors:** Christopher Angelillo, Wing Lam Tock, Matthew Salaciak, Ryan E. R. Reid, Ross E. Andersen, Christine Maheu, Nathalie A. Johnson

**Affiliations:** 1 Department of Kinesiology and Physical Education, Faculty of Medicine and Health Sciences, McGill University, Montreal, Quebec, Canada; 2 Ingram School of Nursing, Faculty of Medicine and Health Sciences, McGill University, Montreal, Quebec, Canada; 3 Department of Medicine, Jewish General Hospital, Montreal, Quebec, Canada; 4 Department of Human Kinetics, St. Francis Xavier University, Antigonish, Nova Scotia, Canada; PLOS ONE, UNITED KINGDOM

## Abstract

**Background and objective:**

Treatments of lymphoma can lead to reduced physical functioning, cancer-related fatigue, depression, anxiety, and insomnia. These side effects can negatively impact the cancer survivor’s quality of life. Mounting evidence indicates that physical activities are highly therapeutic in mitigating the short- and long-term side effects of cancer treatments. Yet, lymphoma survivors’ participation in physical activities remains suboptimal, which has been further exacerbated by the deleterious effects of isolation during the COVID-19 pandemic. The *Lymfit* intervention aims to offer motivational support, expert guidance, and a personalized exercise prescription to optimize physical activities among lymphoma survivors. This proof-of-concept study explores implementation feasibility (retention, technical and safety), and the preliminary effects of *Lymfit* on various health outcomes.

**Method:**

This was a single-armed trial with a pre-and post-test design. Twenty lymphoma survivors were recruited to participate in the 12-week *Lymfit* intervention. Wearable activity trackers (Fitbit) were given to participants as a motivational tool and for data collection purposes. Participants received a personalized exercise prescription designed by a kinesiologist. Physiologic metrics were collected by the Fitbit monitors and were stored in the *Lymfit* database. Self-reported questionnaires measuring health outcomes were collected at baseline and post-intervention.

**Results:**

The retention rate of this trial was 70%. Minimal technical issues and no adverse effects were reported. *Lymfit* led to significant improvements in sleep disturbances and the ability to participate in social activities and decreased fear of cancer recurrence. It also increased daily steps and decreased sedentary time in participants who did not meet the recommended physical activity guidelines.

**Significance:**

With access to resources and fitness centers being limited during the pandemic, the *Lymfit* intervention filled an immediate need to provide physical activity guidance to lymphoma survivors. Findings provide preliminary support that implementing the *Lymfit* intervention is feasible and demonstrated promising results.

## 1. Introduction

Lymphoma is the fifth most common cancer among adults in Canada. It is estimated that approximately 12,000 Canadians were diagnosed with lymphoma in 2021 [1]. There are two main subtypes of lymphoma: Hodgkin’s (HL) and Non-Hodgkin’s lymphoma (NHL), with diffuse large B cell lymphoma [DLBCL]) being the most common NHL. HL and DLBCL are treated with multi-agent chemotherapy with or without radiation [2]. Novel agents, including immune and cellular therapies [3] have markedly improved the cure and remission rates in the relapse setting [4]. These new treatment options have dramatically improved the survival of patients living with and beyond lymphoma [5]. Despite being effective in controlling the disease, these therapies may trigger a variety of negative physical and psychological side effects that can linger post-treatment and significantly affect an individual’s quality of life (QoL), post-treatment.

Treatment-induced toxicity in lymphoma survivors may cause a wide range of short and long term health issues [6]. For instance, radiation treatment to the neck, supraclavicular, and/or mediastinal region increases the risk of radiation-induced hypothyroidism and pulmonary toxicities [7]. Lymphoma survivors are also susceptible to cardiovascular complications owing to the exposure to anthracycline-based regimens and mediastinal/thoracic radiation therapy [8, 9]. Specifically, the risks of developing post-treatment myocardial infarction, arrhythmias, and congestive heart failure among lymphoma survivors are significantly higher than in the general population [10, 11]. Besides the treatment-induced long-term effects, cancer survivors encounter a variety of psychological and functional challenges upon the completion of traditional cancer treatment. These challenges can include anxiety [12], fear of cancer recurrence (FCR) [13], reduced levels of physical functioning [14], cancer-related fatigue [15] and decreased cognitive capability [14], all of which can lead to chronic fatigue and reduced QoL [16, 17].

While researchers have discovered a myriad of health promotion interventions (e.g., dietary or nutritional modification) that can benefit the health of cancer survivors, the positive effects of physical activities (PA) remain one of the most promising options, demonstrating the highest therapeutic value on improved psychological and physical health [18, 19]. In lymphoma patients, moderate to vigorous physical activity (MVPA) pre- and post-treatment are positively associated with various cancer-related outcomes including improved survival [20, 21], improved QoL [22], improved sleep quality [23], higher physical functioning and lower fatigue [24–28].

Despite the overwhelming benefits of PA, creating and implementing lifestyle modifications remains tremendously challenging for cancer survivors [29]. The American College of Sports Medicine (ACSM) suggested exercise interventions designed for cancer survivors should follow the FITT principles [29]: a minimum of 3 times per week (**F**requency); at a moderate-to-vigorous level (**I**ntensity); for 30 minutes each session, for at least 8 to 12 weeks (**T**ime); and, with aerobic activity favoured over resistance training (**T**ype). While the programs should be specific to cancer type, treatments, and/or outcomes, the FITT Principles have been widely adopted in exercise interventions for cancer patients and survivors [30].

Although no exercise intervention studies have been conducted among lymphoma patients in Canada, a structured and supervised exercise intervention was tested among lymphoma patients in Italy and yielded promising results [25]. This Italian in-person intervention was offered in a group format at the oncology institute’s gym, where participants were engaged in 60-minute PA sessions twice a week for eight weeks. The results of the study demonstrated significant improvements in physiological outcomes such as fatigue, body mass index (BMI), flexibility, balance, and functional mobility [25].

While the effectiveness of structured and supervised exercise interventions to mitigate cancer-related side effects have been well established, the 2019 coronavirus disease (COVID-19) pandemic has greatly impacted the format, mode of delivery and implementation of exercise interventions [31]. Since the beginning of 2020, public health measures were imposed by provincial governments in Canada to limit viral transmission. As a result, opportunities to perform PA at health and fitness centers were severely compromised. The home environment emerged as the only viable indoor opportunity for PA, which decreased many cancer survivors’ motivation to engage in PA [32]. Accordingly, the ACSM released a call to action at the beginning of the pandemic for researchers to develop novel and flexible approaches to PA that account for limitations imposed by the pandemic [33].

PA format and modality preferred by cancer survivors during the pandemic include professional guidance, delivered using digital or remote platforms, and home-based programs that offer exercise choices [32]. As reported by lymphoma survivors, the most promising components for supporting PA maintenance include goal setting, accountability, and convenience [34]. In addition, remote interventions using technology such as wearable activity monitors and mobile phones are increasingly being used to incorporate evidence-based components while meeting the expressed desire for convenience and accountability among cancer survivors [35–37].

To date, no intervention aiming to improve PA in individuals with lymphoma during the pandemic has been conducted, neglecting the potentially deleterious effects of the quarantine and sedentary behaviour in this population [38]. These findings underscore the urgent need to develop innovative and enjoyable home-based exercise interventions that promote social distancing, are cost-effective, and have a wide reach to help mitigate the compounding effects of the pandemic on physical inactivity among lymphoma survivors.

Considering this gap in the literature, a research team including members from different disciplines (i.e., kinesiologists, hematologist and information technologists, and nursing scientists) conceptualized and developed an exercise intervention in lights of the increased need for home-based health promotion intervention for lymphoma survivors. The *Lymfit* intervention was developed to remotely deliver a professional guided, tailored exercise prescription, incorporating the use of Fitbit^TM^ monitors aiming to provide support for competency and autonomy in PA engagement, which may translate into improve physiological and psychological health in individuals who live with or beyond lymphoma. The aim of this proof-of-concept (PoC) trial was to explore the implementation feasibility of the intervention by assessing retention rate, technical and safety issues, and the preliminary effects of *Lymfit* on various health outcomes.

## 2. Methodology

### 2.1 Study design

According to the Medical Research Council (MRC) framework [39], the fundamental elements of health intervention development include: engagement with stakeholders (e.g., patient partnership), identification of uncertainties, and continuous refinement of intervention. Hence, the purpose of this PoC trial was to examine whether an intervention is suitable for further testing using a single arm (i.e., single cohort), pre-post-test design. Specifically, this PoC trial aimed to evaluate the feasibility of the design of the 12-week *Lymfit* intervention (i.e., retention rates, technical issues on intervention delivery and data collection during the pandemic, and safety issues) and to explore the preliminary effects of the intervention on study outcomes, including physiologic metrics (i.e., light activity minutes, MVPA, sedentary time, sleep time, and step count) captured by Fitbit^TM^ monitors and three self-reported health outcomes (i.e., QoL, FCR, and fear of COVID). This study was first reviewed by the Research Ethics Board (REB) in October 2019 at the Jewish General Hospital (JGH; Montreal, Quebec) and was approved as a Quality Improvement Program. Due to COVID-related delay, the study was again reviewed and approved by the REB in 2021 (protocol number: 2021–2560). The trial design is guided by the Transparent Reporting of Evaluations with Nonrandomized Designs (TREND) statement checklist [40] (S1 Document) and the reporting of intervention components and procedures is in accordance with the Template for Intervention Description and Replication (TIDieR) checklist [41] (S2 Document). This PoC trial was registered with ClinicalTrial.gov (NCT#05546489). The registering this study was done after enrolment of participants started due to a delay in the administrative procedures. The authors confirm that all ongoing and related trials for this intervention are registered.

### 2.2 Participants, setting, recruitment

This was a single-center study. All study participants were recruited at the Segal Centre, JGH located in downtown Montreal, Quebec. To have been eligible for this study, participants must have met the following inclusion criteria: (1) previously diagnosed with NHL or HL; (2) have completed chemotherapy; (3) have had the approval of their hematologist as having no contra-indications to perform physical activities; and (4) had access to a smartphone or an electronic device (e.g., tablet) that allowed them to attend virtual meetings and to install the Fitbit^TM^ application. The date of their cancer diagnosis and the duration of remission was not a criterion for enrollment in this PoC trial.

**Fig 1:**
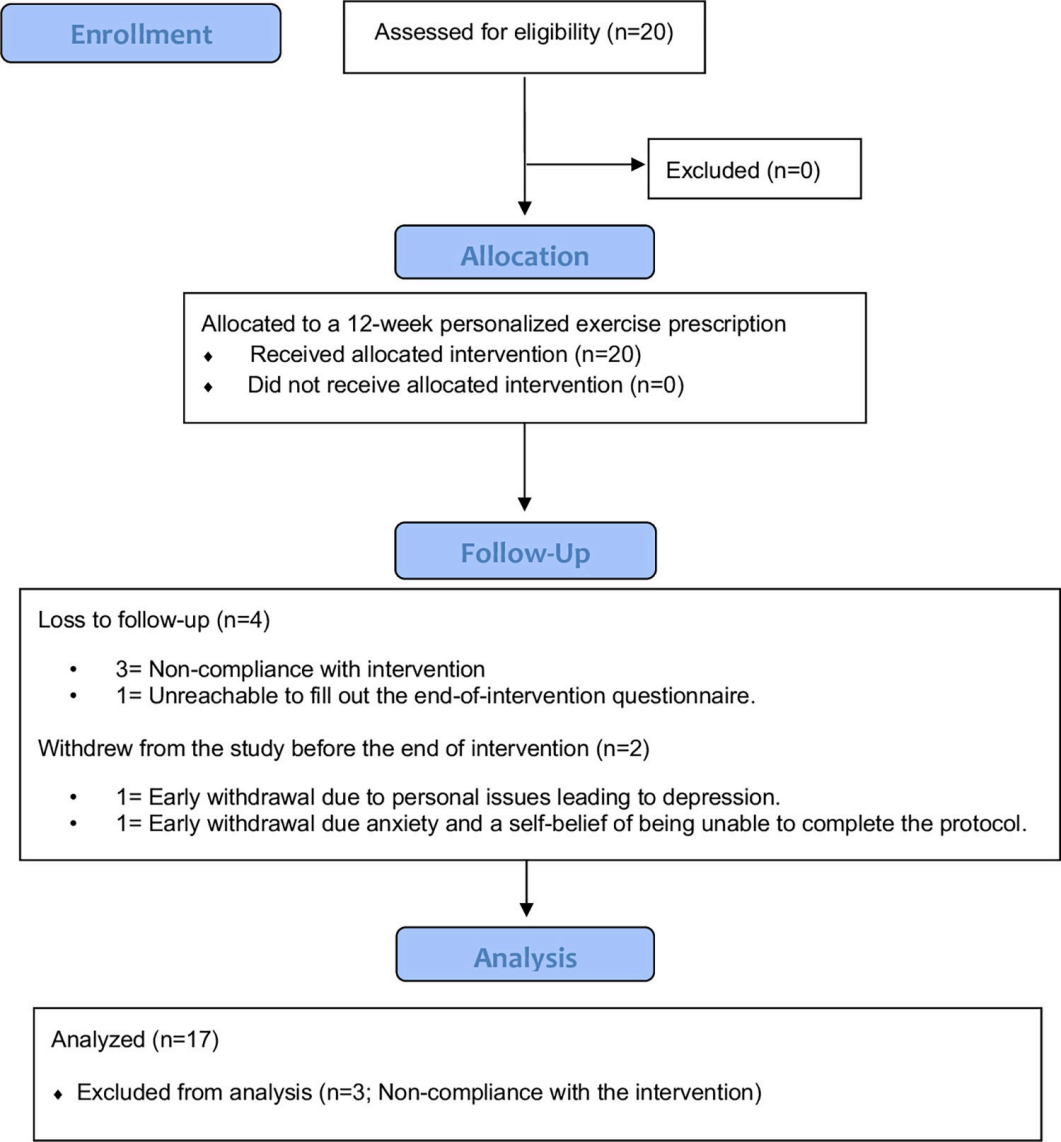
CONSORT flow diagram.

All participants were recruited by their hematologist. Once recruited, participants were contacted by the research coordinator to collect informed consent via videoconferencing. Written informed consent was obtained (electronically via the *Lymfit* platform), witness by the research coordinator, from each participant prior to enrolment. Recruitment commenced in June 2021 and ended in November 2021. Data collection ended in February 2022 when the last recruited participant completed the *Lymfit* intervention. At the time of the data collection, Montreal was in the third wave of COVID-19. A vaccine passport mandate came into effect in September 2021 and a curfew was implemented at the end of December 2021 and lasted until mid-January 2022. The CONSORT flow diagram which has been modified for a non-randomized trial is shown in Fig 1.

### 2.3 Study procedures

After providing consent, the eligible participants completed a set of baseline questionnaires (i.e., demographic + self-reported questionnaires assessing FCR, QoL, and fear of COVID) electronically. All participants were registered to the *Lymfit* platform and assigned a Fitbit^TM^ monitor on the *Lymfit* platform. Each Fitbit^TM^ was mailed to the participant’s home address along with a set of five resistance bands of varying resistance levels.

One important merit of Fitbit^TM^ monitors is that it allows data transfer via Bluetooth technology to the Fitbit^TM^ application program interface (API) through Fitbit^TM^’s smartphone application. The information technology team at the Lady David Institute (LDI) of JGH has developed the *Lymfit* platform on a password-protected secured server. This platform allowed the research team to document participants’ information, and to capture and store participants’ Fitbit^TM^ metrics in a secured database via the Fitbit^TM^ API. This security system ensured full patient confidentiality throughout the study. In addition, participant data were de-identified by assigning each patient’s Fitbit^TM^ device with a code name (e.g., Lymfit001) and an email (e.g., lymfit001@ladydavis.ca), such that the Fitbit^TM^ company had no access to personal identification data.

Once the Fitbit^TM^ monitors and the resistance training bands were received by the participants, the research coordinator met with the participants via videoconferencing to guide the participants and connect their Fitbit^TM^ to their smartphone application using the *Lymfit* study account assigned to them. During the same meeting, the study kinesiologist administered a baseline fitness assessment (S3 Document), which allowed the kinesiologist to design a personalized exercise prescription depending on a variety of self-reported fitness variables (i.e., types of exercise enjoyed/disliked; self-rated stamina, flexibility, coordination, and strength; perceived barriers to PA; exercise preferences and habits). Answers collected in this informal assessment were not included in the data analysis process. Thereafter, participants were instructed to wear the device on the wrist of their non-dominant hand for one week (i.e., seven consecutive days; referred to as week 0) and to maintain their usual level of activity. The data collected in week 0 were considered participants’ baseline activity levels. Considering the information collected in the baseline fitness assessment, and the objective baseline activity level obtained during week 0, the kinesiologist designed a tailored exercise prescription for each participant. At the end of week 0, participants met with the kinesiologist in another videoconferencing meeting to discuss the tailored exercise prescription. An example of an exercise prescription for one of the study participants is shown in S4 Document. The personalized exercise prescription was designed based on the FITT principles suggested by the ACSM Cancer Survivorship exercise guidelines [29] and the participant’s baseline fitness assessment results.

Based on participants’ baseline activity levels and preferences, the kinesiologist encouraged participants to gradually increase their minutes of MVPA. The exercise prescription consisted of both aerobic and resistant training suggested by the FITT principles. For inactive participants or participants who lacked motivation to exercise, the initial goal was to increase the step count. The kinesiologist and participants explored opportunities during the meeting based on the progress in the past two weeks. As these small milestones were achieved, the goal changed to increase the intensity and duration of the activities. For participants who were active at baseline based on current guidelines, the exercise prescription was tailored accordingly. Participants who were successfully achieving 90 min of MVPA/week were encouraged to gradually increase their MVPA to 150 min/week to meet the Canadian general population guidelines [42]. The delivery of the intervention by the graduate kinesiologist student and co-author, CA, was closely supervised by the registered kinesiologist PhD co-author and study team member, RA. Additionally, as part of their participation in the Lymfit study, the student actively contributed to the development of the intervention protocol. During the 12-week intervention, the study kinesiologist followed up with each participant every two weeks to review their progress and to make adjustments to the program where needed. Post-intervention questionnaires assessing self-reported study outcomes administered at baseline were again collected at the end of the intervention. All participants kept the Fitbit^TM^ as a nonmonetary incentive, regardless of whether they successfully completed the intervention. Over the course of the intervention, if a participant’s Fitbit^TM^ has not been synced to the smartphone application for more than 12 hours; taken off their wrist for more than 12 hours; had a low battery level (below 20%), email reminders were sent out to the participants to ensure that the most accurate reading of a participant’s activity throughout each day was attained.

### 2.4 Data collection and outcome measurements

#### 2.4.1 Demographics and clinical characteristics

Demographic variables including age, sex, body mass index (BMI), height, and weight, were collected to broadly encapsulate the characteristics of each participant prior to commencing the intervention. The participant’s primary diagnosis and the time since their last chemotherapy treatment were collected to represent their clinical characteristics.

#### 2.4.2 Implementation feasibility

This outcome was assessed by 1) retention rate, 2) technical issues, and 3) safety (i.e., adverse events). In the bi-weekly meeting with the kinesiologist, participants were instructed to report any adverse events during the intervention. Any technical and safety issues were documented in a study log by the study coordinator and the kinesiologist.

#### 2.4.3 Physiologic metrics

Metrics, including light activity (minutes/day), MVPA (minutes/day), sedentary time (hours/day), total sleep time (hours/day), and steps taken (steps/day) were captured via the Fitbit^TM^ monitors and stored on the *Lymfit* platform. Participants were instructed to sync their Fitbit^TM^ to the smartphone application daily to allow the captured data to be transferred to the *Lymfit* platform.

Fitbits^TM^ are a valid and reliable measure of different physiologic variables, while also being a cost-effective alternative compared to other research-grade activity monitors [43]. A study conducted by Reid and colleagues, compared Fitbit^TM^ monitors to ActiGraph GT3X+ accelerometer, a gold standard in measuring activity in clinical trials, with results indicating that Fitbits^TM^ (particularly those worn on the wrist) were as accurate as ActiGraph GT3X+ in measuring activities, particularly step counts and light activities [44]. It was thus concluded that wrist-worn Fitbit^TM^ monitors are a valid and reliable device for low-intensity activities and steps taken, making them a feasible option for this study.

#### 2.4.4 Self-reported health outcomes

Three self-reported study outcomes were collected using validated instruments at baseline and at 12-week (post-intervention). **QoL** was evaluated using the 29 items Patient-Reported Outcomes Measurement Information System® (PROMIS–29 Profile v2.1) obtained from the PROMIS Health Organization (https://www.promishealth.org). The PROMIS measure perceived health status along seven domains, with items answered on five-point Likert scales. There are four items on each of the following domains: ability to participate in social roles/activities (PROMIS 1), anxiety/fear (PROMIS 2), depression/sadness (PROMIS 3), fatigue (PROMIS 4), pain interference (PROMIS 5), physical function (PROMIS 6), and sleep disturbance (PROMIS 7).

Domain scores were obtained by summing the item scores for each domain. Raw scores generated for each domain will be transformed into a T–score using the scoring service from the Health Measures Assessment Center (https://www.assessmentcenter.net/ac_scoringservice) [45]. For negatively worded domains such as anxiety/fear, depression/sadness, fatigue, pain interference, and sleep disturbance, a higher T-score represents worsening conditions. For positively worded domains such as the ability to participate in social roles/activities and physical functioning, a higher T-score represents improving conditions [46]. The reliability and construct validity of the PROMIS -29 v2.1 were supported in a previous study of cancer survivors [47].

FCR was assessed using the Fear of Cancer Recurrence Inventory (FCRI) [48]. The FCRI is a multidimensional, 42-item questionnaire measuring seven factors pertaining to FCR: triggers, severity, psychological distress, coping strategies, functioning impairments, insight and reassurance. Each item is rated on a five-point Likert scale ranging from *‘not at all’ or ‘never’ (0*) to *‘a great deal’ or ‘all the time’ (4)*. A subscale score can be calculated by summing the item scores of each factor subscale. The total score (ranges from 0 to 168) is then calculated based on the scores of each subscale. Considering that the question for item 13 (“I believe that I am cured and the cancer will not come back”) is addressed in the opposite direction of other questions, the response scale to item 13 is reversed before calculating the total score. A higher summary score of FCRI indicates higher levels of FCR. In addition, the nine-item severity subscale of the FCRI has an empirically validated cut-off score (≥ 13 points) for screening clinically significant levels of FCR [49]. Psychometric properties of the English version of FCRI including internal consistency and test–retest reliability have been confirmed in mixed cancer survivors [50].

Fear related to the pandemic was assessed using the Fear of COVID scale (FCV-19S) [51], a seven-item scale assessing the fear of COVID–19. The items are rated on a five-point Likert scale ranging from *“strongly disagree” (1)* to *“strongly agree” (5)* with total scores ranging from 7 to 35. Higher total scores represent higher levels of fear. The reliability and validity of the FCV-19S have been established [51]. All data described in sections 2.4.3 to 2.4.4 are available at the following Open Science Framework (OSF) link: osf.io/j4yau [52].

### 2.5 Data analysis

Descriptive statistics were used to summarize the basic demographic and physical characteristics of study participants. For the feasibility outcomes, the retention rate was calculated as a percentage of the total number of those initially enrolled, and any technical and safety issues were reported narratively.

Physiologic metrics were reported using descriptive statistics, including frequency, percentage, mean, and standard deviation (SD; expressed in mean ± SD in the rest of the paper). For all metrics captured by the Fitbit^TM^ (i.e., light activity, MVPA, sedentary time, total sleep time, and steps taken), the daily averages were computed (i.e., the mean of a seven-day period from Monday to Sunday) from week 0 to week 12. This allowed the investigators to observe changes in Fitbit^TM^ metrics on a week-to-week basis. To account for missing data, a day with a daily step count less than the pre-determined cut-off of 1000 steps/day was excluded from the daily average over a week (seven days) as it was assumed that the participant did not wear the Fitbit that day. The day was included in the seven-day average only if the participant had more than 1000 steps in the day. This cut-off was determined based on a previous study by Craig et al. (2010) [53], which stated that values <1000 daily steps should be considered outliers and may be removed from analyses to trim the data, and to ensure that days in which the participants may not have properly worn their Fitbit^TM^ was not included in the analyses. A cut-off point for four out of seven days was also required for that week to be considered a valid daily average. If there are less than four valid days of data for that particular week, that week of data for that participant was omitted in the analysis. This threshold was determined based on the guidelines established by Trost et al. (2005) [54], which states that between three and five days of objective monitoring is required to estimate physical activity outcomes in adults reliably. If a participant had more than four invalid weeks out of the 12 weeks, they were considered a lost to follow-up.

For the three self-reported health outcomes, data was first screened for normality of distributions using the Shapiro-Wilk Test. Given the lack of normality, a nonparametric (two-sided) Wilcoxon Signed-Rank test was conducted to compare and determine if there were statistically significant within-group changes among the scores in the three self-reported health outcomes (i.e., PROMIS, FCRI, and Fear of COVID) from pre- to post-intervention. The effect size estimate r for non-parametric test was calculated by converting the z-score with the equation r = z/N [55] and was interpreted using Cohen’s guidelines for r of 0.1 = small effect, 0.3 = medium effect, and 0.5 = large effect [56]. Data analysis was performed using R version 4.2.0 (R Core Team, Vienna, Austria) and a was set to 0.05.

## 3. Results

### 3.1 Participant characteristics

The baseline demographic characteristics of the 17 participants are presented in Table 1. The mean age of this study cohort was 31.5 ± 7.3 years, 58.8% were female (n = 10) and 53% of the study participants’ BMI was above 25 (n = 9). Regarding participant’s clinical characteristics, 76.5% (n = 13) were diagnosed with HL and 76.5% (n = 13) had completed their treatment (i.e., chemotherapy) over one year ago, ranging from one to six year.

**Table 1:** Baseline demographic and clinical characteristics of participants (N = 17).

Variables	mean	sd	n	%
Age (years)	31.5	7.3		
Height (cm)	170.4	9.8		
Weight (kg)	75.7	18.8		
BMI				
Healthy (18.5–24.9)			8	47.1
Overweight (25–29.9)			7	41.2
Obese (30+)			2	11.8
Sex				
Male			7	41.2
Female			10	58.8
Diagnosis				
HL			13	76.5
DLBCL			4	23.5
Time since chemotherapy completion				
> 1 year (range: 1 to 6 years)			13	76.5
< 1 year			4	23.5

Note: Sex, BMI, diagnosis and time since diagnosis are expressed as n (%); Age, Height, and Weight are expressed as Mean (SD).

### 3.2 Implementation feasibility

The retention rate of this PoC trial was 70%. Twenty participants consented and were enrolled in this study. Of these, 14 participants completed the *Lymfit* intervention, including the post-intervention questionnaires. Two participants withdrew from the study at weeks three and 10. The reasons given by the participants for discontinuation from the study included personal issues leading to depression, and a self-belief that they would not be able to participate consistently for the duration of the study. One participant did not complete the post-intervention questionnaire and was considered lost to follow-up. With the participant’s permission, Fitbit^TM^ data collected from the three participants up to the time of withdrawal/ end of intervention were retained and analyzed.

Another three participants’ Fitbit^TM^ data were excluded from the analysis. The three participants had stopped wearing their Fitbit ^TM^ and were unresponsive to our multiple reminders. Therefore, the data collected from these participants were considered invalid (i.e., the participants wore the Fitbit^TM^ for less than four days per week, and for over four weeks during the intervention). In terms of demographic and clinical characteristics, the three excluded participants (including two males and one female) have completed chemotherapy more than one year ago. The mean BMI of the three participant was 27.1, which is categorized as overweight.

Overall, 17 participants’ Fitbit^TM^ data were included in the analysis. Technical issues were noted in this study. For instance, resistance and strength training was not accurately tracked, which could lead to potentially inaccurate minutes spent in MVPA. In addition, an error with electronic questionnaire delivery was noted with one participant, which was resolved by the *Lymfit* technical support staff immediately upon detection of the issue. Finally, no adverse effects or other safety issues were reported by the study participants, except for one participant who reported a mild skin irritation induced by a metal (nickel) piece attached to the Fitbit^TM^ monitor.

### 3.3 Physiologic metrics

Fitbit^TM^ metrics including light activity, MVPA, sedentary time, total sleep time, and steps taken were reported in a daily average to determine the activities of this cohort at baseline (week 0). Mean light activity at baseline was 217.9 ± 94.7 minutes per day. MVPA was 21.1 ± 14.2 minutes per day. It is important to note that at baseline, 76.5% (n = 13) of participants met the recommended weekly PA guideline for cancer survivors (i.e., 90 minutes of MVPA per week) [29]. Interestingly, the mean sedentary time at baseline was 13.8 ± 2.7 hours, which far exceeded the daily recommended sedentary behaviour guidelines of less than eight hours of sedentary time [42]. The mean sleep time (i.e., total time spent asleep) for this population was also lower than the recommended guidelines, with a baseline of 6.6 ± 1 hour per day and only 35.3% (n = 6) met the recommended sleep guidelines of seven to nine hours per night [42] at baseline. Finally, the mean daily steps taken at baseline was 8144 ± 3616 steps per day.

During the intervention period, Fitbit metric values from baseline to week 12 were analyzed. Favourable changes were noted for mean light activity minutes, which increased slightly from 217.9 ± 94.7 minutes per day at baseline to 223.2 ± 92.3 minutes per day at week 12, an increase of 5.3 minutes per day. Moreover, the mean sedentary time decreased slightly from 13.8 ± 2.7 hours per day to 13.6 ± 3.5 hours per day, and the mean sleep time showed a substantial increase from 6.6 ± 1 hour per day to 7.1 ± 1.2 hours per day.

However, less favourable changes were noted, whereby there was a slight decrease in mean MVPA from 21.1 ± 14.2 minutes per day at baseline to 17.5 ± 16.4 minutes per day at week 12, while mean light activity minutes increased slightly from 217.9 ± 94.7 minutes per day to 223.2 ± 92.3 minutes per day. There was also a slight decrease in the mean daily steps taken from 8144 ± 3616 steps per day to 7231.8 ± 2872.2 steps per day, a decrease of approximately 912 steps per day.

**Fig 2:**
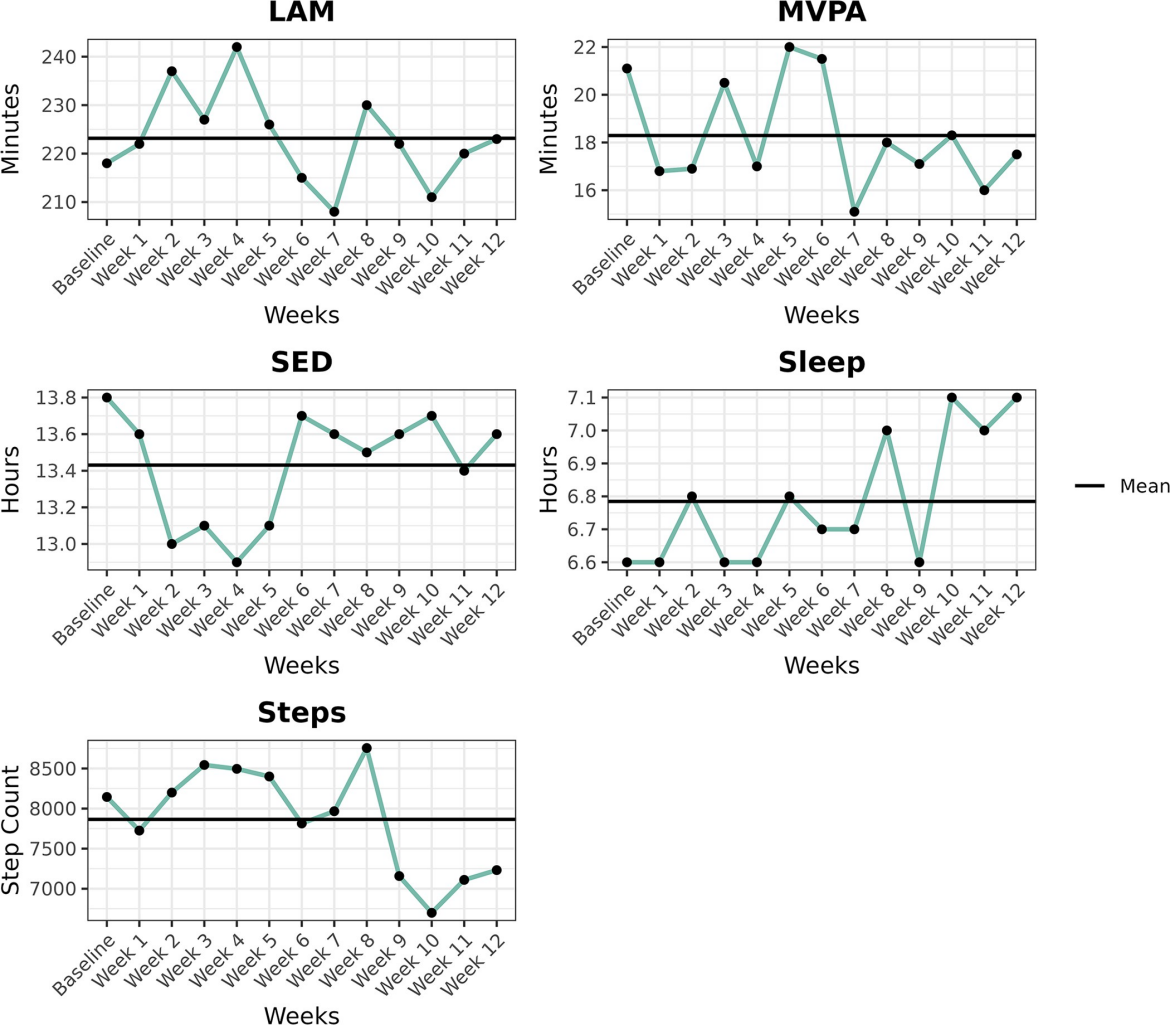
Changes from baseline to 12 weeks across all physiologic metrics. Note: LAM = light activity minutes (mins/day); MVPA = Moderate to vigorous intensity physical activity (mins/day); SED = sedentary time (hours/day), Sleep = total sleep time (hours/day); Steps = steps taken (steps/day).

As shown in Fig 2, the mean light activity increased from baseline to approximately the halfway mark of the study, then gradually declined until the end of the study. It ranged from a high of 242.1 ± 81 minutes at week four to a low of 208.5 ± 91.2 minutes at week seven. The daily average time spent in MVPA fluctuated from baseline throughout the 12 week-intervention, ranging from a high of 22 ± 16.4 minutes at week five to a low of 15.1 ± 13.3 minutes at week seven. Mean sedentary time fluctuated from the baseline, though each week during the intervention reported lower mean sedentary times than baseline. It ranged from a high of 13.7 ± 3.4 hours at week six to a low of 13 ± 2.5 hours at week four. Mean sleep time steadily increased from the baseline, ranging from a high of 7.1 ± 1.4 hours at week 12 and a low of 6.6 ± 1.2 hours at week nine. Finally, the mean steps per day fluctuated from the baseline as well, ranging from a high of 8755.3 ± 4250.4 at week eight of intervention to a low of 6699 ± 2468 at week 10.

Of the four participants (23.5%) who did not meet the recommended PA guidelines for cancer survivors at baseline, all four increased their daily average MVPA from baseline to week 12. At baseline, these four participants averaged a mean of 4.5 ± 5.7 minutes of MVPA. This increased to 10.2 ± 16 minutes at week 12. Similar improvements were made in mean sedentary time. At baseline, these participants averaged 13.4 ± 2.3 hours of sedentary time, which decreased to 12.9 ± 2.4 hours at week 12. Similarly, the mean light activity increased from 178.2 ± 111.3 minutes at baseline to 215.9 ± 116.8 minutes at week 12. Finally, the mean daily steps taken saw a sharp improvement in these four participants as well, which increased from 4312 ± 2425 at baseline to 5584 ± 2170.2 at week 12.

### 3.4 Self-reported health outcomes

**Fig 3:**
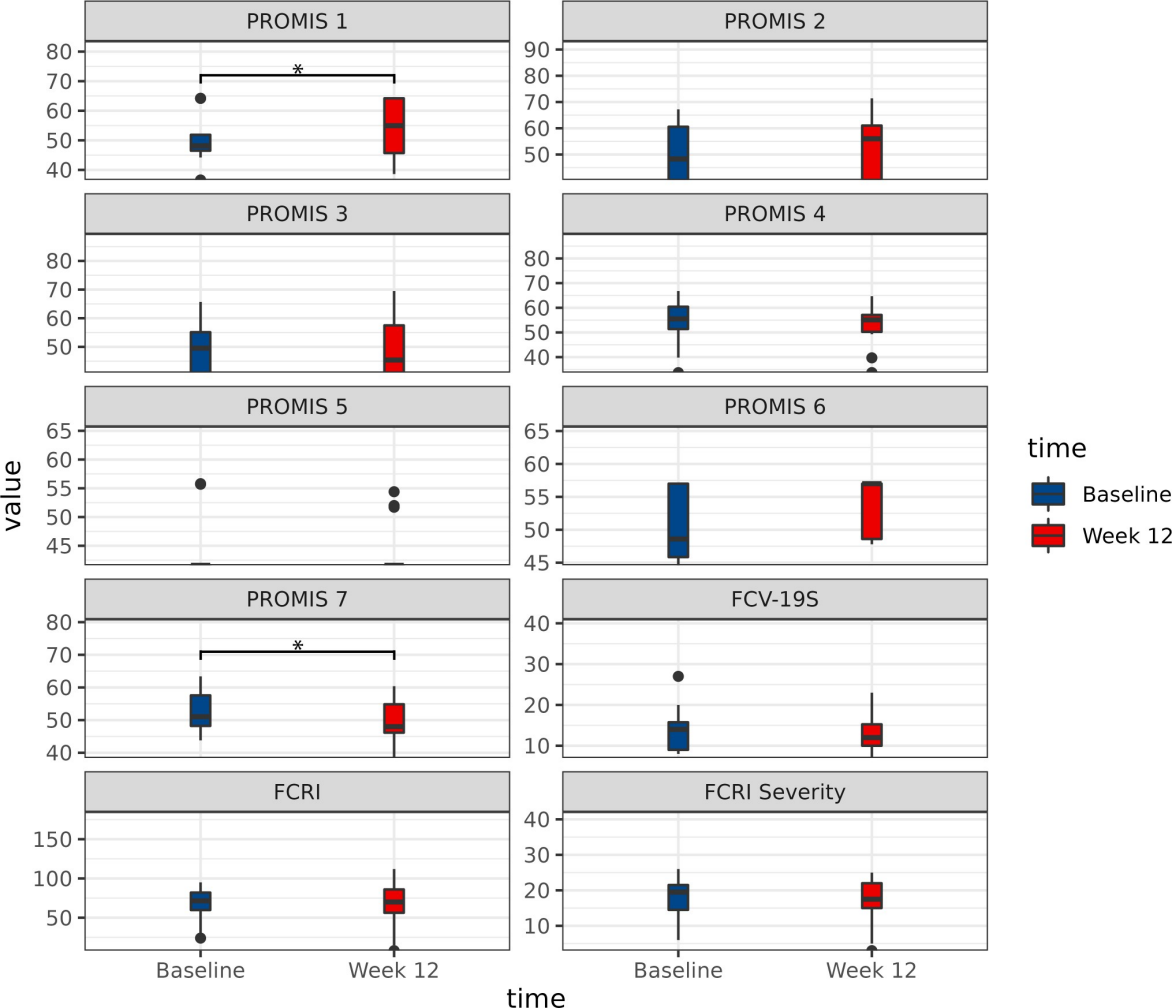
Comparisons between baseline and 12 weeks across all self-reported health outcomes.

Wilcoxon signed–rank tests were used to examine the pre- and post-intervention changes in self-report outcomes. The results including medians (Mdn), effect sizes (ES), z-scores, and *p*-values are displayed in Table 2 and Fig 3. Of the seven QoL domains measured by PROMIS 29, analysis results revealed that three domains had a significant change in scores from pre- to post-intervention. PROMIS 1 (ability to participate in social roles/activities) scores improved significantly from pre-intervention (Mdn = 48.15) to post-intervention (Mdn = 54.95; Z = − 2.01, *p* = 0.044), signifying a significant overall improvement in their ability to participate in social activities. In addition, participants scored statistically significant lower on PROMIS 7 (sleep disturbance) from pre-intervention (Mdn = 51.05) to post-intervention (Mdn = 48.0; Z = 0.76, *p* = 0.045), signifying lowered sleep disturbance after the intervention.

**Table 2:** Self-reported health outcomes baseline and 12-week comparisons.

Variables ^a^	Mdn at baseline (Min–Max) ^b^	Mdn at 12-week (Min -Max)	Effect size (interpretation)		Z score	*p*-value ^c^
PROMIS 1	48.15 (36.60–64.20)	54.95 (38.60–64.20)	0.54	(large)	-2.01	0.044 *
PROMIS 2	48.30 (40.30–67.20)	56.00 (40.30–71.40)	0.14	(small)	-0.53	0.593
PROMIS 3	49.55 (41.00–65.70)	45.40 (41.00–69.50)	0.02	(small)	0.08	0.932
PROMIS 4	55.50 (33.70–66.80)	55.10 (33.70–64.70)	0.30	(medium)	1.13	0.255
PROMIS 5	41.60 (41.60–55.80)	41.60 (41.60–54.40)	0	/	0	1.0
PROMIS 6	48.60 (44.60–57.00)	57.00 (47.80–57.00)	0.45	(medium)	-1.69	0.090
PROMIS 7	51.05 (43.80–63.40)	48.00 (38.40–60.40)	0.53	(large)	2.00	0.045 *
FCRI	71.50 (24.00–95.00)	70.00 (8.00–112.0)	0.20	(small)	0.76	0.442
FCRI Severity	19.50 (6.00–26.00)	17.50 (3.00–25.00)	0.50	(large)	1.90	0.057
FCV-19S	14.00 (8.00–27.00)	12.00 (7.00–23.00)	0.31	(medium)	1.16	0.246

^a^ PROMIS = Patient-Reported Outcomes Measurement Information System®; PROMIS 1 = ability to participate in social roles/activities; PROMIS 2 = anxiety/fear; PROMIS 3 = depression/sadness; PROMIS 4 = fatigue; PROMIS 5 = pain interference; PROMIS 6 = physical function; PROMIS 7 = sleep disturbance; FCRI = Fear of Cancer recurrence Inventory; FCV-19S = Fear of COVID Scale

^b^ Mdn = Median; Min = minimum; Max = Maximum

^c^ p-values are obtained using two-sided Wilcoxon Signed-Rank test

* p < 0.05

Results revealed that FCRI Severity decreased from pre-intervention (Mdn = 19.5) to post-intervention (Mdn = 17.5; Z = 0.76, *p* = 0.057). While the resulted p-value did not reach conventional levels of statistical significance, the large effect size (ES = 0.50) indicate that participants had lowered FCR immediately post-intervention. It is important to note that this cohort of participants reported a clinical level of FCR (≥ 13 points), both pre- and post-intervention. However, the total FCRI score had no significant changes from pre- to post-intervention (*p* = 0.442).

No significant differences were detected in median scores for the other four QoL domains of the PROMIS-29 questionnaire, which include PROMIS 2 (*p* = 0.593), PROMIS 3 (*p* = 0.932), PROMIS 4 (*p* = 0.255), PROMIS 5 (*p* = 1.0), and PROMIS 6 (*p* = 0.090) indicating that there were no significant reductions in anxiety/fear, depression/sadness, fatigue, pain interference, or physical function. Finally, results showed that while participant’s reported FCV-19S score decreased from baseline (Mdn = 14.00) to post-intervention (Mdn = 12.00), these changes were not significant (Z = 1.16, *p* = 0.246), indicating that there was also no significant reduction in participant’s fear of COVID immediately post-intervention.

## 4. Discussion

The primary aim of this PoC trial was to determine the implementation feasibility of delivering a remote exercise intervention during the COVID-19 pandemic by assessing the retention rate, as well as technical and safety issues. Determining the preliminary effects of the intervention on physiological and health outcomes also informs the significance of the intervention on the health and QoL of lymphoma survivors.

The main finding of the present PoC trial was the successful implementation and delivery of the *Lymfit* exercise intervention among lymphoma survivors during the COVID-19 pandemic. The retention rate of this study was considered acceptable at 70% when compared to the 10–42% average attrition rate estimated for clinical trials of exercise interventions for cancer patients in the literature [57]. Although there were a few minor technical issues encountered by the study team, they were resolved by the *Lymfit* technical support staff. This finding is consistent with previous literature that has also demonstrated the feasibility of delivering a home-based exercise intervention during the COVID-19 pandemic among high-risk individuals [58]. However, to our knowledge, exercise interventions have not been conducted through which physiologic data was measured objectively among lymphoma survivors (i.e., from Fitbit^TM^ metrics). This presented an additional challenge of ensuring that the technology used in this study (Fitbit^TM^ and *Lymfit* platform) worked properly in unison and was feasible for use in a home-based exercise intervention. This PoC trial proved how technology can be integrated into an exercise intervention to measure and track participants’ fitness and activity outcomes objectively. This PoC trial confirmed that the *Lymfit* platform is ready to be implemented in the next phase of intervention testing (i.e., a pilot study with a randomized controlled trial [RCT] design).

Overall, QoL also improved among the participants. The total sleep time among this cohort had shown an upward trend from baseline to week 12. Participants who were already meeting the seven to nine-hour daily sleep guidelines for adults at baseline continued to meet the guidelines throughout the entire study. A gradual increase was seen in those who averaged less than seven hours of sleep per day at baseline. Given our small sample size and the absence of an increase in PA, we cannot conclusively state, as reported in the literature, that the improvement in sleep quality among the lymphoma survivors studied was solely attributable to PA engagement. However, it is important to note that this result is consistent with previous research highlighting the association between PA and reduced sleep disturbances and improved sleep quality in cancer patients and survivors [59, 60].

Among the three self-reported health outcomes, significant improvements were observed among some domains relating to QoL, including the ability to participate in social rolesand sleep disturbance. This is consistent with previous literature, which has shown that performing PA post-treatment can significantly improve domains related to QoL in cancer patients, including social role satisfaction [61]. Improvements in sleep disturbances have also been reported in a previous trial involving cancer patients, which concluded that patients who reported sleep disturbances due to chemotherapy could improve their quality of sleep for three to six months upon completion of an exercise intervention [62]. It is important to note that the observed improvements in QoL may have been influenced by other factors, such as the social support provided by the kinesiologist or other unmeasured influences.

Although FCR severity had decreased slightly immediately following the completion of the intervention, the severity levels, both pre- and post-intervention, were above the cut-off for clinical FCR. This finding indicates exercise alone may not be sufficient to diminish the psychological effects of cancer and its treatments. Psychoeducational interventions provided at the end of cancer treatment have shown promising results in mitigating the level of FCR for cancer survivors [63]. Future studies can incorporate additional psychoeducational strategies to mitigate anxiety about cancer recurrence among lymphoma survivors.

The physiologic metrics (i.e., light activity minutes, MVPA, sedentary time, sleep time, and step count) captured by Fitbit^TM^ monitors did not show obvious improvements. This contradicts previous research that has indicated that a home-based exercise intervention can significantly improve fitness outcomes, particularly MVPA, among a sedentary population [64]. This finding can be attributed to the fact that the *Lymfit* study cohort was already moderately active at baseline. Most of the study participants met the PA guidelines at baseline (week 0). The daily average MVPA of this sample at baseline exceeded the recommended PA guidelines for cancer patients of 90 minutes per week [29]. Similarly, the baseline daily step average of 8144, while not quite meeting the recommended guidelines of 10000 steps per day, was relatively high and placed this sample in the “somewhat active” category [65]. However, of the participants who did not meet the recommended PA guidelines at baseline (i.e., less than 90 minutes of MVPA at baseline), all of them were able to improve their physiologic metrics, including increased weekly MVPA, increased step counts and decreased sedentary time. This indicates the benefits of a home-based exercise intervention on individuals who are considered sedentary, which will be more representative of the participants that will be recruited in the next phase (i.e., a pilot RCT) of this study due to more precise inclusion criteria as to the period that participants must have completed chemotherapy within the last six month. Importantly, the intervention also helped those who were already adequately active maintain their levels of activity.

Despite being moderately active, sedentary minutes among the study cohort at baseline were high and remained high throughout the intervention. This may be explained by the Fitbit^TM^ monitor’s inability to accurately record movements that do not sustain an elevated heart rate such as short-distance walking, which also overestimates sedentary time. In line with the literature, a recent study has reported that Fitbit^TM^ monitors can overestimate sedentary time by an average of 37 minutes a day in adults of a healthy weight range, which is likely due to inaccurate classification of some light activity as sedentary time [66]. The pandemic and the restrictions that followed may also explain this observation. This study was conducted at the height of the 3rd wave of the COVID-19 pandemic, where physical distancing, lockdowns and home quarantines were either mandated or strongly encouraged. These restrictions led to steep declines in PA and increases in sedentary time among adults in the general population [67]. Therefore, an intervention designed to reduce sedentary behaviours would likely have benefitted this cohort much more than one designed to increase PA. This type of intervention could involve assisting participants in setting goals designed to reduce sedentary behaviours (e.g., cannot surpass “x” number of hours of sedentary time per day), as well as discussing the risks of increased sedentary time with them.

This PoC trial should be interpreted within the context of important strengths. Firstly, this study was among the first to demonstrate that a remote exercise intervention can be implemented safely and effectively during the COVID-19 pandemic in a high-risk population. Preliminary evidence of the intervention was promising. The intervention shows a potential to improve QoL outcomes among individuals affected by lymphoma during the pandemic; an unprecedented time where cancer survivors’ lifestyle habits have been dramatically altered [68]. Given the remote design of this intervention, patients were not required to travel to a specific location to exercise outside of their own homes. This was significant as avoiding public areas and the risk of COVID-19 infection was the priority throughout the intervention. Secondly, this study was among the first to implement Fitbit^TM^ monitors to track and collect physiologic metrics for analysis. To our knowledge, no study had previously implemented activity monitors to track the PA of lymphoma patients post chemotherapy. The implementation of such technology opens a variety of different ways that fitness data can be prescribed, interpreted and analyzed. Further, Fitbits^TM^ are cost-effective and can be easily adapted into clinical practice, thus benefiting all cancer survivors completing their treatments [36]. Lastly, *Lymfit* was uniquely positioned to provide a remotely delivered exercise intervention during the pandemic, which was in time to address the immediate needs of lymphoma survivors. There are growing issues that the lockdown and the social distancing restrictions imposed by the provincial governments have limited opportunities for people to be physically active [67]. Further, the general population increased their sedentary time and reduced their PA levels during quarantine, contributing to controversial psychological outcomes [69]. Cancer patients and survivors are disproportionally impacted by the pandemic [70, 71]. With uncertainty regarding post-pandemic PA environments and behaviours, the *Lymfit* intervention may help foster clinically meaningful improvements in lymphoma survivors’ MVPA and QoL. Indeed, a major advantage of *Lymfit* is that it is a remote intervention that can benefit any patient with access to a smart phone. This increases the potential outreach to patients by including those living in remote settings, far from a fitness center or those who want to continue social distancing to decrease their risk of infections.

Several limitations within this study should also be noted. Firstly, the sample size of this PoC trial may have been too small to make inferences regarding QoL and fitness metrics. Given the large variability in the physiologic metric data, the increasing or decreasing trend should be interpreted with caution. However, due to the pilot nature of this trial, it was important to maintain a small-scale study cohort and not prolong the pilot phase of the study. Secondly, this PoC trial was a single-site study, meaning participants were recruited solely from the JGH in Montreal. The COVID-19 pandemic and subsequent restrictions limited opportunities to expand the trial to multiple sites, thereby limiting the generalizability of the results. Furthermore, the single cohort pre-and post-test design impeded the ability to draw initial intervention effectiveness conclusions. The results of this study should be interpreted with caution, given the large variations within the data. This was inevitable and unavoidable in a PoC trial with a small participant pool. Thirdly, as a PoC trial, participants’ baseline PA levels and their differences in exercise motivation were not considered to be an inclusion criterion for the intervention. Further, individuals who view PA as important are more likely to participate in interventions such as *Lymfit*. These pre-existing differences among lymphoma survivors may have led to potential selection bias, which can be confounders and may have potentially influenced the study observations. Future trials might benefit from certain screening procedures to select patients who are less active, engaged in more sedentary behaviours, or lack exercise motives to participate in the intervention. Finally, most of the study participant’s time since treatment completion was over one year, which may have inadequately captured the effects of PA during the critical early survivorship period.

The PoC trial proves that the *Lymfit* intervention can be remotely delivered. Preliminary results also indicate that this intervention has the potential to improve QoL in lymphoma survivors. If proven feasible and effective in future testing, the *Lymfit* intervention would provide healthcare professionals with a healthy alternative to mitigate the toxic effects of medication, which may improve their patient’s short- and long-term QoL. Furthermore, it would provide healthcare professionals with a broader outreach and allow them to treat patients living long distances from treatment centers or patients who want to avoid public spaces and potential exposure to COVID-19 or other infectious diseases. In the next testing phase, it is suggested that lymphoma patients who are still undergoing treatment or immediately post-treatment should be included. Twhis will allow researchers to determine if a personalized exercise intervention is effective in promoting QoL and physical fitness in this population. The intervention would also benefit from further refinement guided by a behavioral change theory.

## 5. Conclusion

The scientific evidence is constantly consolidating the crucial and positive impacts of regular PA in cancer in reducing recurrence and improving both short and long-term side effects. This PoC trial established the practicality and implementation feasibility of *Lymfit*, a virtual, personalized exercise intervention that is timely and valuable during the unprecedented circumstances of the pandemic. Promising trends in physiologic metrics and improvements in several self-report health outcomes have been noted in the study results, demonstrating the preliminary success of *Lymfit* in improving the health and well-being of lymphoma survivors. The uncertainties and limitations identified in this PoC trial provide valuable information for the research team to refine the intervention before any further testing. Future trials examining the *Lymfit* intervention with an experimental design and for other health outcomes are warranted.

## Supporting information

S1 Document Transparent Reporting of Evaluations with Nonrandomized Designs (TREND) checklist. (DOC)

S2 Document Template for Intervention Description and Replication (TIDieR) checklist. (DOCX)

S3 Document Baseline fitness assessment. (DOCX)

S4 Document Weekly exercise prescription sample. (DOCX)

S1 Protocol (DOCX)
