# Supplementary material for: A single-armed proof-of-concept study of Lymfit: A personalized, virtual exercise intervention to improve health outcomes in lymphoma survivors in the pandemic
Source: PLoS One. 2024 Jan 5;19(1):e0275038. doi: 10.1371/journal.pone.0275038 (PMC10769060; doi:10.1371/journal.pone.0275038)
Supplement: S3 Document — (DOCX) [file pone.0275038.s003.docx]

**Supplementary document 3. Baseline Fitness Assessment**

Name:

Height: Weight: Waist Circumference:

1. How often were you participating in physical activity prior to diagnosis?
2. 4 to 5 times per week
3. 2 to 3 times per week
4. 1 to 2 times per week
5. Not at all
6. Are you currently involved in regular exercise? Yes No
7. What sport or activity has worked for you in the past?
8. What type of exercise do you enjoy the most?
9. What type of exercise do you dislike and why?

Rate yourself on a scale of 1 (least fit) – 10 (most fit) for each fitness category:

1. How good is your stamina?
2. How strong do you think you are?
3. How flexible do you think you are?
4. How coordinated do you think you are?
5. How much time are you willing to devote to an exercise program?

_____Min/Day ____Days/Week

1. Are there any barriers that may prevent you from exercising on any given day? (e.g., lack of time, family obligations, lack of motivation…)
2. What days and/or time (morning, afternoon or night) are you available to exercise throughout the week?
