# Supplementary material for: A single-armed proof-of-concept study of Lymfit: A personalized, virtual exercise intervention to improve health outcomes in lymphoma survivors in the pandemic
Source: PLoS One. 2024 Jan 5;19(1):e0275038. doi: 10.1371/journal.pone.0275038 (PMC10769060; doi:10.1371/journal.pone.0275038)
Supplement: S4 Document — (DOCX) [file pone.0275038.s004.docx]

| Time/ Period | Monday | Tuesday | Wednesday | Thursday | Friday | Saturday | Sunday |
| --- | --- | --- | --- | --- | --- | --- | --- |
| Morning | **Resistance Training**  **Lower Body**  Using the red resistance band:  1. 1.5 squats: 30 seconds 3:20  2. 20-30 second rest  3. Off-set reverse lunge: 30 seconds each leg 3:17.  4. 20-30 second rest  5. Standing hip abductions: 30 seconds each leg 1:53  6. 20-30 second rest  7. Single leg Romanian deadlift: 30 seconds each leg 2:55.  8. 20-30 second rest  9. Tip-toe side-walks :30 seconds 3:37.  10. Repeat the circuit (numbers 1-9) 3 more times. Rest 60-90 seconds before each circuit. | **Cardiovascular Endurance**  30-min selected cardio exercise* at moderate pace  OR  20-min home high-intensity interval training | **Resistance Training**  **Upper Body**  Using the yellow or red resistance band:  1. Banded shoulder press (right arm): 30-45 seconds 0:26  2. Rest 15 seconds  3. Banded shoulder press (left arm): 30-45 seconds 1:25  4. Rest 15 seconds  5. Pulse raises: 30-45 seconds 2:31  6. Rest 15 seconds  7. Triceps extension (right arm): 30-45 seconds 3:36  8. Rest 15 seconds  9. Triceps extension (left arm): 30-45 seconds 4:40  10. Repeat the circuit (1-9) once more. Rest 60-90 seconds before starting the circuit over. After you have completed the circuit twice, rest 60-90 seconds then move on to 11.  11. Single arm row (right arm): 30-45 seconds 11:01  12. Rest 15 seconds  13. Single arm row (left arm): 30-45 seconds 12:15  14. Rest 15 seconds  15. Single arm alternating lat pull downs: 30-45 seconds 13:15.  16. 15 second rest  17. Single arm bicep curl (right arm): 30-45 seconds 14:17  18. 15 second rest  19. Single arm bicep curl (left arm): 30-45 seconds 15:25  20. Repeat the circuit (11-19) once more. Rest 60-90 seconds before starting the circuit over.  *For this workout, rest in between exercises can be increased if needed. | **Cardiovascular Endurance**  30-min selected cardio exercise* at moderate pace  OR  20-min home high-intensity interval training | **Resistance Training**  **Core**  Using the yellow or red resistance band:  1. Ab Bicycles: 30 seconds 0:55  2. Rest 15 seconds  3. Plank jacks: 30 seconds 1:44  4. Rest 15 seconds  5. Ab marches: 30 seconds 2:27  6. 15 second rest  7. Plank side crunches: 30 seconds 3:12  8. 15 second rest  9. Switch kicks: 30 seconds 3:58  10. 15 second rest  11. Knee tucks: 30 seconds 4:40  12. Repeat the circuit (1-11) 3 more times. Rest 60-90 seconds between each circuit.  *For this workout, rest in between each exercise can be increased if needed. | Rest | Rest |

* Some cardio exercise options include but are not limited to walking, jogging, running, biking, swimming, stairclimbing, rowing, elliptical exercise, etc.

Workout video 1: <https://www.youtube.com/watch?v=87Vh2NcTjbc>

Workout video 2: <https://www.youtube.com/watch?v=wwkrup_TG6I>

Workout video 3: <https://www.youtube.com/watch?v=ggCvel0I__Q>

Workout video 4: <https://www.youtube.com/watch?v=fBLHhD8iX3g>
