# Supplementary material for: A single-armed proof-of-concept study of Lymfit: A personalized, virtual exercise intervention to improve health outcomes in lymphoma survivors in the pandemic
Source: PLoS One. 2024 Jan 5;19(1):e0275038. doi: 10.1371/journal.pone.0275038 (PMC10769060; doi:10.1371/journal.pone.0275038)
Supplement: S1 Protocol — (DOCX) [file pone.0275038.s005.docx]

**TITLE:** **Lymfit to improve fitness and quality of life in lymphoma survivors: A PROOF-OF-CONCEPT Trial**

**PRINCIPAL INVESTIGATORS**:

Nathalie A. Johnson, MD, PhD, FRCPC

Christine Maheu, RN, PhD

**CO-INVESTIGATORS**:

Ross Andersen, PhD

Kelly Davison. MD

**SOURCE OF FUNDING:** Ride to Conquer Cancer

**1.0. Introduction and Background**

The most common cancer in adolescents and young adults (YA, age 18-39) is lymphoma. Lymphoma is a cancer of the immune system treated with chemotherapy with or without radiation. While 60-80% of patients are cured, the treatment isn’t without toxicity. Throughout their therapy, patients can experience nausea, fatigue, insomnia and anxiety. Some may also have more serious side effects, such as infections or neuropathy. Once the cancer is in remission, the patient may still experience many symptoms that prevent them from returning to work or school, due to changes in weight, hair, deconditioning, fear of cancer recurrence and prolonged fatigue. Exercise has been shown to improve many symptoms in cancer patients. This has led to revised evidence-based fitness guidelines for cancer patients and survivors. Recommendations are to perform 30 minutes of moderate to vigorous physical activity (MVPA) three times per week (1). The most profound and significant benefit is in reducing fatigue, whereas the effect on other cancer symptoms is less clear. Furthermore, the role of exercise in reducing cancer related symptoms has been mainly studied in patients with breast and prostate cancer and not YA patients with lymphoma.

**2.0. Literature Review**

**2.1. Current Standard of Care:** Lymphoma is the 6th most common cancer in Canada and the most common cancer diagnosed in YA, with Hodgkin lymphoma (HL) and diffuse large B-cell lymphoma (DLBCL) being the main subtypes (2). Over 80% of patients are cured following the 4-6 months administration of multi-agent chemotherapy. More specifically, ABVD (doxorubicin, bleomycin, vinblastine and dacarbazine), is given once every 2-weeks to HL patients and RCHOP (rituximab, cyclophosphamide, doxorubicin, vincristine and prednisone) is given every 3-weeks to DLBCL patients. These drugs have considerable toxicities, which include cardiac and pulmonary toxicity, peripheral neuropathy and an increased risk of developing infections from immune suppression. Moreover, the administration of radiation therapy in combination with doxorubicin to early-stage HL and some DLBCL patients significantly increases the long-term risk of CVD. Patients who achieve a complete remission enter a “follow up” phase, where they are typically monitored with blood tests and physical exams every 3-months for 2 years, which is the period where the risk of relapse is highest. They are then seen every 6-months for 3 years and yearly thereafter.

**2.2. Patient Concerns and Relevance of Research Question:** While health care providers’ main concerns during the chemotherapy phase is toxicity (*e.g*., infections) that could delay treatment and compromise cure, the patients’ concerns are extreme fatigue, nausea, anxiety, insomnia, pain and poor **health related quality of life (hrQOL)** (3,4). Although these short-term complications are partially addressed with medications, including steroids, anti-emetics, anxiolytics, analgesics and sedatives, they too are not without their side-effects. In the follow-up phase, **fear of cancer recurrence** can be disabling, requiring referrals to psychologists in some patients (5).

**2.3. FITT Guidelines for Exercise in Cancer Survivors:** It is universally accepted that exercise reduces the risk of obesity, type II diabetes (DM2), osteoporosis and small vessel disease (SVD) in the general population. As such, the World Health Organization recommends that adults should engage in a minimum of 150 minutes of moderate to vigorous physical activity (MVPA) per week (6). While these guidelines were initially extended to cancer survivors, field experts have since reviewed the literature and recently published evidence-based guidelines for cancer survivors during and post-chemotherapy called the FITT guidelines (1). These guidelines recommend that cancer survivors perform exercise a minimum of 3-times per week (**F**requency), at moderate to vigorous physical activity (MVPA) (**I**ntensity), 30-min each session, for at least 8-12 weeks (**T**ime), with aerobic activity favored over resistance training (**T**ype). Compliance with these guidelines has demonstrated improvement in fatigue, anxiety, depressive symptoms, hrQOL and physical function in cancer survivors. Taken together, exercise may be a good intervention for lymphoma patients to minimize both short and long-term complications of therapy.

**2.4. Rationale and Knowledge Gaps:** Encouraging lymphoma patients to exercise during therapy is **not** routinely recommended by hematologists in Quebec. The primary focus during treatment is disease control while limiting toxicity (mainly infections), with the understanding that fatigue and anxiety will eventually subside with time. The international exercise guidelines are primarily based on studies performed in patients with breast and prostate cancer. These studies relied heavily on activity self-reporting, which have their limitations from relying on memory of activities done in the past or overinflated accounts from patients wanting to portray themselves in a socially acceptable manner (7) Thus, further studies using *methods that can accurately* determine if exercise is associated with improved bio-psycho-social health in lymphoma survivors are needed.

**3.0. Hypothesis and Objectives:**

The primary objective of this proof-of-concept study is to determine the feasibility of the Lymfit intervention for future application in a larger trial. The purpose is then to determine if a personalized fitness program, with the help of a fitness tracker (Fitbit), can improve their activity levels, quality life, and well-being. This proof-of-concept study also aims to examine any potential safely-issues related to the remotely delivered exercise program.

**4.0 Research Plan:**

**4.1. The Lymfit Intervention:** Both supervised training (*i.e.,* a coach) and activity trackers (*e.g.,* Fitbits) has great potentials improve adherence to exercise prescriptions (8,9). Each participant will be given a 12-week exercise program, which will be constructed based on the recommendations from the FITT guidelines for cancer survivors by a kinesiologist. The participants in the study will follow up with the kinesiologist every two weeks to discuss any necessary changes within the exercise prescription.

**4.2. Methodology:** This single arm proof-of-concept trial will be a phase one study of a larger randomized controlled trial (RCT). Ethics approval will be obtained from the Research Ethics Board (REB) at the Jewish General Hospital (JGH; Montreal, Quebec). Participants in the study will be prescribed a personalized exercise program for 12-weeks, and their physical activity will be monitored using a FitBit. We have recently developed “Lymfit”, a server that allows our team to retrieve and store data from Fitbit devices on a secure network at the JGH. Physical parameters including activity minutes, daily step counts, sleep time, sedentary minutes, will be collected from the participant’s Fitbit and are stored in the Lymfit server. To preserve patient confidentiality, we will de-identify patient data by assigning each patient’s Fitbit device with a name (*e.g.* Lymfit001) and an email (*e.g.* Lymfit001@ladydavis.ca), such that Fitbit has no access to personal patient data other than the date of birth, height and weight. The participants will complete a set of study questionnaires at **baseline, and at 12-weeks** (post-intervention).

**4.3. Participants and Recruitment:**

***Inclusion criteria:*** 1) lymphoma patients who received initial diagnosis at the age of age 18-39, 2) approved by their hematologist as having no contra-indications to vigorous exercise, 3) receiving or has received chemotherapy with curative intent, 4) own a cellular phone that is able to download the Fitbit app and to complete the study questionnaires online, and 5) is comfortable with teleconferencing with at least one platform (Zoom or Teams).

***Exclusion criteria:*** 1) The patient has contra-indications to vigorous exercise, or 2) the patient doesn’t want to wear a fitness tracker due to various concerns as no Fitbit data would be collected.

***Number of subjects:*** This is a proof-of-concept study to determine if the implementation of the exercise program is feasible and acceptable in this patient population. A total of 20 participants will be recruited to participant in this study.

***Recruitment*:** They will be recruited by their hematologist at the JGH. The JGH treats 20-25 newly diagnosed HL and DLBCL/year, with both sexes represented ~equally.

**4.4. Procedures:**

After the hematologist briefly discusses the project with the patient, interested participants will be contacted by a research coordinator who will explain the study in detail and obtain informed consent. The research coordinator will register the participants on the Lymfit server and enter baseline demographics. Each participant will fill out the first set of questionnaires and be given an assigned Fitbit (e.g. Lymfit 001) and will be registered at the Fitbit server as Lymfit001@ladydavis.ca. For this study, we will use the Fitbit Inspire tracker, which will be synced with the Fitbit App and downloaded to their phone. All Fitbits can retrieve the fitness data required for this study, the Inspire tracker was chosen because it was within the scope of this proposal’s budget. Data from the Fitbit needs to be synced to the Fitbit server at least twice weekly (the data stored on the device for 5 days). The data will be transferred to our secure server at the JGH every night. This server is protected by a firewall such that only the study investigators have access to the patient data.

We will ask patients to perform their baseline activity assessment during the first week with their Fitbit. After that first week, the kinesiologists will examine the baseline physical activity and sedentary behaviours of the patient and schedule an appointment with him/her using teleconferencing (e.g. zoom) due to the physical distancing measured imposed by the covid19 pandemic. The default intervention in the Fitbit tracker is to track and motivate participants to increase their activity each week until they reach the guidelines set for cancer patients (90 min/week). They will be given personalized instructions for exercise prescriptions based on their baseline fitness level and exercise history at baseline, and every two week-there-after (given by teleconferencing). For instance, for sedentary patients or symptomatic patients that lack any motivation to exercise, the initial goal would be to increase the step count and to decrease sitting time. The kinesiologist and patient can explore opportunities throughout the day to take a series of brief walks. As these small milestones are achieved, the goal would be to increase the duration of activity (more steps) and to increase the intensity of the activity (i.e. increase the heart rate to be considered “active” minutes by increasing the pace of walking or by walking up stairs). In more active patients, exercise will be tailored accordingly, and will not require that the patient engage in these activities at a gym, where exposure to potentially infectious contacts is increased. In motivated patients who have successfully achieved 90 min of MVPA/week, we will encourage them to gradually increase their activity to 50 min x 3 times/week to meet the Canadian general population guidelines (16). For the purpose of this study, Fitbit data will be collected from 0 to 12-weeks during the exercise intervention. Patients will be contacted by email if there is no activity detected from their Fitbit for over 12 hours and when the battery level is too low.

**4.5. Data Collection:**

Feasibility outcomes will be collected and documented throughout the study by the research coordinator. The Lymfit server will store the Fitbit metrics, questionnaires responses will be collected at baseline, and at 12 weeks.

**4.5.1. Feasibility:**

This outcome will be assessed by 1) retention rate, 2) technical issues, and 3) safety (i.e., adverse events). In the bi-weekly meeting with the kinesiologist, participants will be instructed to report any adverse events during the intervention. Any technical and safety issues will be documented in a study log by the study coordinator and the kinesiologist.

**4.5.2. Fitbit Metrics:**

Metrics, including light activity (minutes/day), MVPA (minutes/day), sedentary time (hours/day), total sleep time (hours/day), and steps taken (steps/day) will be captured via the Fitbit trackers and stored on the Lymfit server. Participants will be instructed to sync their Fitbit to the smartphone application daily to allow the captured data to be transferred to the Lymfit server.

**4.5.3. Self-Reported Health Outcomes:**

*Quality of Life:* QoL will be evaluated using the 29 items Patient-Reported Outcomes Measurement Information System® (PROMIS–29 Profile v2.1) obtained from the PROMIS Health Organization (<https://www.promishealth.org>) (10). The PROMIS measure perceived health status along seven domains, with items answered on five-point Likert scales. There are four items on each of the following domains: ability to participate in social roles/activities, anxiety/fear, depression/sadness, fatigue, pain interference, physical function, and sleep disturbance. PROMIS measures have been validated in >5000 people with lymphoma and other cancers, has robust psychometric properties and has been calibrated specifically in a large population-based cohort of people recently diagnosed with lymphoma and other cancers, and cancer patients beginning an exercise program (11,12).

*Fear of Cancer Recurrence (FCR):* The Fear of Cancer Recurrence Inventory (FCRI) was developed to assess the multidimensional aspects of FCR. The FCRI consists of seven subscales: triggers, severity, psychological distress, coping strategies, functioning impairments, insight, and reassurance. The 42 items are rated on a 5-point Likert scale ranging from 0 to 4. The FCRI is a reliable and valid self-report scale (13).

*Fear of Covid 19:* Fear related to the pandemic will be assessed using the Fear of COVID scale (FCV-19S) (14), a seven-item scale assessing the fear of COVID–19. The items are rated on a five-point Likert scale ranging from “1: strongly disagree” to “5: strongly agree” with total scores ranging from 7 to 35. Higher total scores represent higher levels of fear.

**5.0. Data Analysis:**

Descriptive statistics will be used to provide a summary of the basic demographic and physical characteristics of study participants. For the feasibility outcomes, the retention rate will be calculated as a percentage of the total number of those who are initially enrolled, and any technical and safety issues will be documented by the study coordinator. Physiologic metrics will be reported using descriptive statistics (i.e., frequency, percentage, mean, standard deviation). For all metrics captured by the Fitbit (i.e., light activity, MVPA, sedentary time, total sleep time, and steps taken), the daily averages will be computed from week 0 to week 12.

Missing data: Any day with a daily step count less than the pre-determined cut-off of 1000 steps/day will be excluded from the daily average over a week. A day will be included in the seven-day average only if the participant had more than 1000 steps in the day. A cut-off point for four out of seven days will also be required for that week to be considered a valid daily average. If there are less than four valid days of data for that particular week, that week of data for that participant will be omitted in the analysis. If a non-compliant participant had more than four invalid weeks out of the 12 weeks, they will be considered a lost to follow-up.

**6.0. Funding**:

Funding for the Lymfit database and server, as well as the Fitbits, is provided by Dr. Johnson’s funds raised during the 2019 and 2018 *Ride to Conquer Cancer*. Funding for the students are provided by the investigator’s private funds. Further funding will be sought in 2021.

**7.0. Risk and Inconvenience:**

There exists the remote possibility during vigorous exercise of adverse changes including, but not limited to, abnormal blood pressure, fainting, dizziness. There also exists mild risk of physical injuries related to exercising outdoors. These risks will be minimized by tailored instructions based on the patients’ baseline activity levels and preferences. The increase in activity will be gradual and only safe activities will be promoted during this project. Furthermore, patients who have baseline co-morbidities that may be at higher risk of injury would be excluded based on their hematologist’s assessment.

There is a theoretical risk of breach in confidentiality if someone is capable of hacking into the Lymfit server located at the Lady Davis Institute of the JGH. However, it is protected by firewalls such that that nobody, except for the investigators on the study, will have access to the data, by logging on with a unique username and password. Furthermore, the data and downstream analysis will only be used using codes and no patient identifiers will be used. Each participant will have a research ID, which will be the Fitbit ID (i.e. Lymfit 001). This coded data will protect the patient from having the Fitbit company use their personal data (only date of birth, height and weight will be provided). At the end of the study, the raw Fitbit data (with patient identifiers) will be kept on the server for 2 years, after which point it will be stored on a hard drive in Dr. Johnson’s office, which will not be accessible to anyone but Dr. Johnson. Coded data will be available to the other investigators.

**8.0. Benefits:**

The patients will be able to access their own data in real time on their Fitbit device and phone, which will motivate them to become more active. Furthermore, they can consent to have their own results of this study as well as the final study results, an option that is available on the consent form. This research has the potential to directly and immediately benefit patients by improving hrQOL, cardiovascular fitness and if sustained, decrease long-term risk factors for cardiovascular disease, diabetes and osteoporosis. Given the benefits of exercise in the general population and other cancer survivors, we anticipate that the Fitbit intervention will increase physical activity and fitness in lymphoma survivors and that this will translate into an improved QOL and reduce the fear of cancer recurrence.

**9.0. Compensation:**

# Participants will be provided with Fitbits pedometers, which they will keep even at the end of the project. This is to ensure that there is no infectious contamination between participants if these devices were to be re-used. Participants won’t be financially compensated for their time or transportation. All interventions will be done remotely (e.g., by zoom) to minimize social contact and risk of Covid transmission.

**10.0. Incidental Findings:**

We don’t anticipate there to be incidental findings in this project. Patients will have access to their data in real time via their Fitbit device and Fitbit App on their phone. If the study reveals extremely high levels of anxiety or depression, or if the Fitbit data reveals heart rates in the abnormal range, this information will be relayed to the treating hematologist. This disclosure is stated on the consent form. The hematologist has access to multiple health care professionals that can help with manage symptoms, including a nurse, a psychologist, a social worker, a pharmacist and a nutritionist. Furthermore, he/she could consult other physicians as needed (e.g. a cardiologist, psychiatrist…).

**11.0. Communication and publication of results:**

The results of this research will be communicated to the treating hematologists and health care workers that are involved in the care of lymphoma survivors. This will hopefully improve the outcome of future lymphoma survivors. The research will also be presented at meetings and conferences by any of the investigators on the study.

**References:**

1. Campbell KL, Winters-Stone KM, Wiskemann J, May AM, Schwartz AL, Courneya KS, et al. Exercise Guidelines for Cancer Survivors: Consensus Statement from International Multidisciplinary Roundtable. Med Sci Sports Exerc. 2019 Nov;51(11):2375–90.

2. Canadian Cancer Statistics. Canadian Cancer Statistics - Canada.ca 2019 [Internet]. Canadian Cancer Statistics. 2019 [cited 2019 Aug 21]. Available from: <https://www.canada.ca/en/public-health/services/chronic-diseases/cancer/canadian-cancer-statistics.html>

3. Sodergren SC, Husson O, Rohde GE, Tomasewska IM, Vivat B, Yarom N, et al. A Life Put on Pause: An Exploration of the Health-Related Quality of Life Issues Relevant to Adolescents and Young Adults with Cancer. J Adolesc Young Adult Oncol. 2018 Mar 22;7(4):453–64.

4. Macpherson CF, Hooke MC, Friedman DL, Campbell K, Withycombe J, Schwartz CL, et al. Exercise and Fatigue in Adolescent and Young Adult Survivors of Hodgkin Lymphoma: A Report from the Children’s Oncology Group. J Adolesc Young Adult Oncol. 2015 Sep;4(3):137–40.

5. Sun H, Yang Y, Zhang J, Liu T, Wang H, Garg S, et al. Fear of cancer recurrence, anxiety and depressive symptoms in adolescent and young adult cancer patients. Neuropsychiatr Dis Treat. 2019 Apr 8;15:857–65.

6. World Health Organization. Physical activity and adults: Recommended levels of physical activity for adults aged 18 - 64 years. 2020. [cited 2020 Oct 16]. Avaliable from: https://www.who.int/dietphysicalactivity/factsheet_adults/en/

7. Nadler MB, Desnoyers A, Langelier DM, Amir E. The Effect of Exercise on Quality of Life, Fatigue, Physical Function, and Safety in Advanced Solid Tumor Cancers: A Meta-analysis of Randomized Control Trials. J Pain Symptom Manage. 2019 Nov;58(5):899-908.e7.

8. Singh B, Zopf EM, Howden EJ. Effect and feasibility of wearable physical activity trackers and pedometers for increasing physical activity and improving health outcomes in cancer survivors: A systematic review and meta-analysis. J Sport Health Sci. 2021. doi: 10.1016/j.jshs.2021.07.008.

9. Clifford BK, Mizrahi D, Sandler CX, Barry BK, Simar D, Wakefield CE, et al. Barriers and facilitators of exercise experienced by cancer survivors: A mixed methods systematic review. Support Care Cancer. 2018;26(3):685-700. doi: 10.1007/s00520-017-3964-5.

10. Gershon RC, Rothrock N, Hanrahan R, Bass M, Cella D. The use of PROMIS and assessment center to deliver patient-reported outcome measures in clinical research. J Appl Meas. 2010;11(3):304-14. Epub 2010/09/18. PubMed PMID: 20847477; PubMed Central PMCID: PMCPMC3686485.

11. Musanti R, Chao Y-Y, Collins K. Fitness and quality of life outcomes of cancer survivor participants in a community exercise program. J Adv Pract Oncol. 2019 Feb;10(1):24–37.

12. Hartman SJ, Weiner LS, Nelson SH, Natarajan L, Patterson RE, Palmer BW, et al. Mediators of a physical activity intervention on cognition in breast cancer survivors: Evidence from a randomized controlled trial. JMIR Cancer. 2019 Oct 11;5(2):e13150.

13. Custers JAE, Gielissen MFM, Janssen SHV, de Wilt JHW, Prins JB. Fear of cancer recurrence in colorectal cancer survivors. Support Care Cancer. 2016 Feb;24(2):555-562. doi: 10.1007/s00520-015-2808-4. Epub 2015 Jun 25. PMID: 26108170; PMCID: PMC4689743.

14. Ahorsu DK, Lin C-Y, Imani V, Saffari M, Griffiths MD, Pakpour AH. The fear of COVID-19 scale: Development and initial validation. Int J Ment Health Addict. 2020. doi: 10.1007/s11469-020-00270-8.
